# Child maltreatment and management of pediatric patients during COVID-19 pandemic: Knowledge, awareness, and attitudes among students of medicine and surgery. A survey-based analysis

**DOI:** 10.3389/fpubh.2022.968286

**Published:** 2022-09-20

**Authors:** Giovanni Aulino, Flavia Beccia, Michele Rega, Chiara Siodambro, Giuseppe Capece, Stefania Boccia, Antonio Lanzone, Antonio Oliva

**Affiliations:** ^1^Department of Health Surveillance and Bioethics, Section of Legal Medicine, Fondazione Policlinico Universitario A. Gemelli IRCCS, Università Cattolica Del Sacro Cuore, Rome, Italy; ^2^Section of Hygiene, University Department of Life Sciences and Public Health, Università Cattolica del Sacro Cuore, Rome, Italy; ^3^Università Cattolica del Sacro Cuore, Fondazione Policlinico Universitario A. Gemelli IRCCS, Rome, Italy; ^4^Department of Woman and Child Health and Public Health—Public Health Area, Fondazione Policlinico Universitario A. Gemelli IRCCS, Rome, Italy; ^5^Unit of Obstetrics and Obstetric Pathology, Department of Woman and Child Health and Public Health, Fondazione Policlinico Universitario A. Gemelli IRCCS, Rome, Italy

**Keywords:** child maltreatment, child safety, medical liability, medical education, telemedicine, COVID-19, survey

## Abstract

**Purpose of the study:**

To assess perception, awareness, and attitudes regarding the medico-legal relevance of child maltreatment and management of pediatric patients during the COVID-19 pandemic in a cohort of medicine and surgery students, with a particular focus on child safety and maltreatment.

**Methods:**

A cross-sectional, web-based survey was conducted through an anonymous questionnaire on the personal websites of Università Cattolica del Sacro Cuore medical students.

**Results:**

The study included 1,166 participants, the majority of whom were experienced with child maltreatment and defensive medicine; only a small percentage was aware of the government's efforts to prevent child maltreatment and safeguard vaccination physicians. Moreover, there was no agreement on the use of telemedicine for non-serious pediatric patients or on the consequences it might have on their health. Finally, the detrimental impacts of lockdown on children's mental health are a major worry.

**Conclusions:**

Knowledge of these themes is mainly implemented by deepening these concepts during the undergraduate studies since a high level of knowledge on child maltreatment and on the management of COVID-19 pandemic was significantly associated with clinical years of course. Specific seminars analyzing telemedicine and legislative protections concerning minors and those concerning vaccination doctors should be included in the study plan to raise awareness these concepts.

## Introduction

The first coronavirus disease (COVID-19) outbreak was announced at the end of December 2019 in Wuhan, China. The virus soon spread globally, and, for this reason, the World Health Organization declared a state of pandemic (1).

The first country that was affected in Europe was Italy. On January 31, 2020, the Italian government declared the state of emergency and established a number of measures aimed at limiting the spread of the virus.

The first nationwide lockdown was imposed on March 3, 2020, following the isolation of the first affected municipalities. Restrictions were implemented based on the regional spread of the virus until March 31, 2022 (2–4).

Many concerns have been raised about how these measures, particularly the lockdown, may affect the health, especially the mental health, of the most vulnerable individuals, including children.

Governments have taken steps to lessen the economic effects of the COVID-19 pandemic, but there has not been an adequate response to reduce domestic violence, child abuse and mental health deterioration (5). Added to this is the impact on the mental health of healthcare professionals who have been on the frontline during the pandemic and the constant risk of professional liability (6).

Doctors and medical and surgical students are probably ill-equipped to deal with the pandemic's effects on children's mental health and physician professional responsibility in the coming years.

For these reasons, the aim of this study was to assess how a cohort of medical students perceived, were aware of, and felt about the medico-legal relevance of these themes in the context of the COVID-19 pandemic, with a focus on child abuse and the treatment of pediatric patients.

## Materials and methods

### Study design and participants

A cross-sectional, web-based survey was conducted during May 2022, in the period immediately following the end of the State of Emergency. Through outlook form, an anonymous questionnaire was administered on the personal websites of medical students attending the undergraduate course at the Università Cattolica del Sacro Cuore. As this survey was addressed to medical students, the study was based on a non-probability, voluntary sample. Participation was voluntary and unpaid.

### Questionnaire design

The questionnaire (see Supplementary File 1) consisted of various sections covering the following aspects: demographic data (gender, age, course year and region of origin), knowledge and awareness of child maltreatment and, finally, the aspects of patient management during the COVID-19 pandemic. In total, the questionnaire comprised 23 questions. Students were asked to answer the questions using both binary answers and a 5-point Likert scale.

### Validation of the questionnaire

To further improve the study's quality, a two-step external validation process was used. First, a heterogeneous team of students and professors from various disciplines conducted cognitive pretesting on a small sample size of 8, and then a larger cohort of 50 medical students from the course of medicine and surgery underwent pilot testing (7, 8).

### Statistical analyses

The reliability of the scales was measured using the Cronbach's alpha coefficient. Using 1,000 boot-strap samples, the 95% confidence interval (CI) for each alpha value was estimated. All variables were then subjected to descriptive analyses.

Using multivariable logistic regression models, factors of knowledge and awareness on the two issues of concern (management of the COVID-19 pandemic and child abuse) were analyzed. The two tiers of the two demographic variables—region of residence and year of course—were combined from the original numerous categories.

Regions were recoded into Center-North and South and Islands and year of course was recoded into preclinical years (1–4) and clinical ones (5 and 6). Respondents were judged to have a high degree of expertise when they correctly answered 75% of the included questions. Only dichotomic questions were included. Age, gender, course year, and region of residence were all covariates that were accounted for in the models. Multivariable logistic regression models were built using the Hosmer and Lemeshow method (9).

Each variable underwent univariable analysis using the proper statistical test (logistic regression model), and when the *p*-value was <0.15, it was added to the multivariable logistic model.

The influence of the independent variables on each binary outcome investigated was expressed as odds ratios (OR) and 95% confidence interval (CI).

All statistical analyses were performed using Stata software, version 14 (StataCorp LP, College Station, TX).

### Ethical considerations

To participate, students had to give their informed consent. The study protocol was approved by the Ethics Committee of the Policlinico Universitario A. Gemelli IRCCS.

## Results

### Participants' demographic

Out of a total of 1,833 students, 1,183 questionnaires (64.54%) were collected. Seventeen of the latter were excluded because they did not provide informed consent or had incomplete information. Thus, 1,166 (98.56%) questionnaires were included in our results.

Table 1 provides a description of the response rate divided by course year. In general, a higher response rate was observed in the last 3 years compared to the first.

**Table 1:** Number of responses per course year.

**Course year**	**Number of** **answers**	**Total number** **of students**	**Percent (%)**
1	152	299	50.83
2	152	295	51.53
3	187	269	69.52
4	189	270	70
5	249	286	87.06
6	237	414	57.25
Total	1,166	1,833	63.61

### General characteristics of students

**Figure 1:**
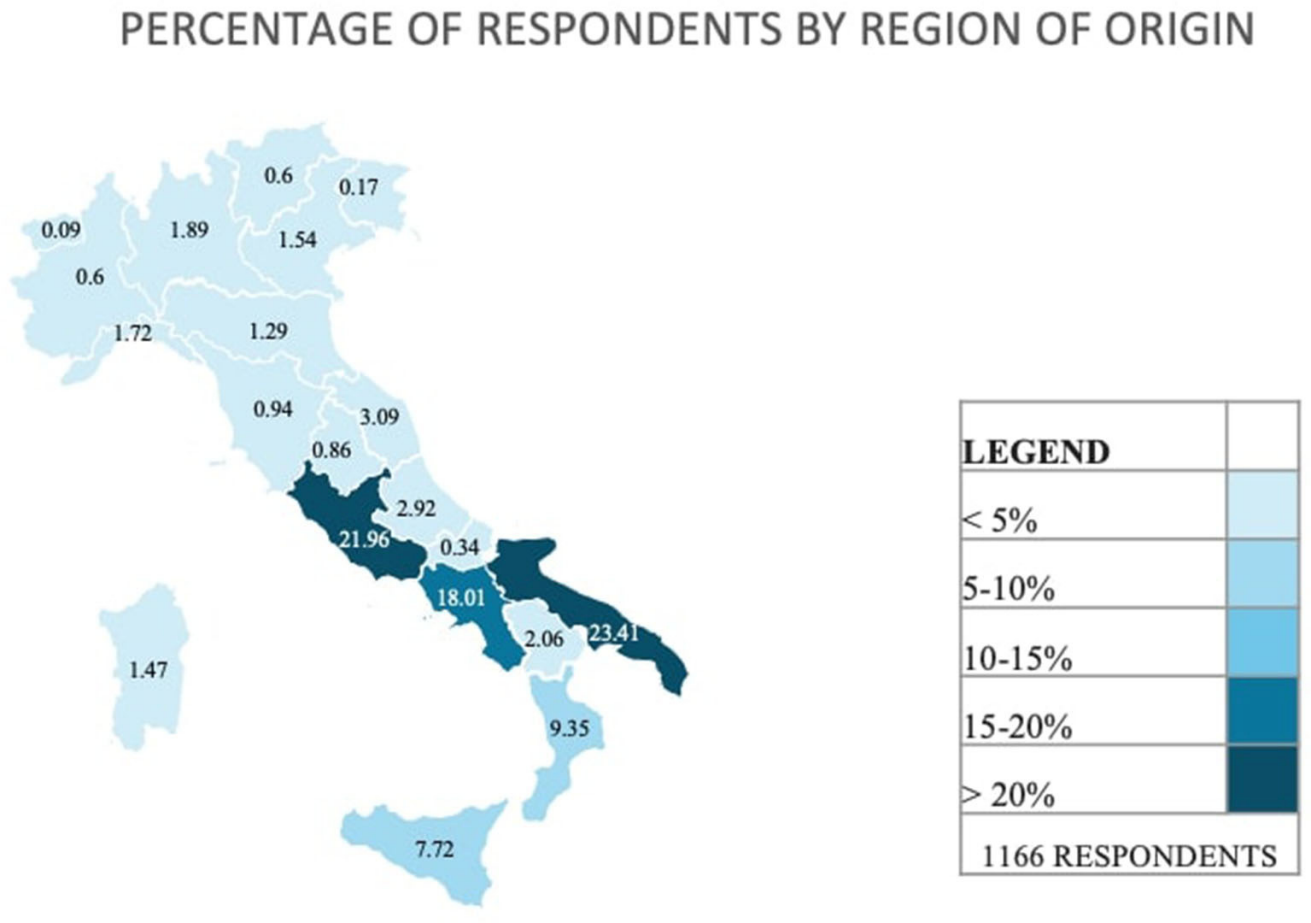
Map of percentages of respondents by region of origin.

Table 2 provides information on the demographic characteristics of the participants. The median age of participants was 23.04 years (IQR 22–24), and females accounted for 52.74% of the total. Around 42% of the respondents attended the 5th and 6th year of course (21.36 and 20.33%, respectively). Most students were resident in Southern Italy (62.35%) and the most representative regions were Apulia, Latium, and Campania (23.41, 21.96, and 18.01% respectively) (Figure 1).

**Table 2:** Student responses to the questionnaire administered.

**Variable**	**Category**	**Number**	**Percent (%)**
**General characteristics of students**			
Age	Median 23.04 (S.D. 1.80, IQR 22–24)		
Gender	Male	551	47.26
	Female	615	52.74
Year of course	1	152	13.04
	2	152	13.04
	3	187	16.04
	4	189	16.21
	5	249	21.36
	6	237	20.33
Area of origin	Southern regions	654	56.09
	Center regions	313	26.85
	Islands	107	9.19
	Northern regions	92	7.9
Area of residence	Center-North	439	37.65
	South and Islands	727	62.35
**Focus on child maltreatment**			
Knowledge of child maltreatment situations	Yes	607	52.06
	No	559	47.94
Knowledge about the increase in child maltreatment cases during the pandemic	Yes	1,107	94.94
	No	59	5.06
Expecting an increase of child maltreatment cases in the future	Yes	1,049	89.97
	No	117	10.03
Knowledge of government measures issued child maltreatment	Yes	136	11.66
	No	1,030	88.34
Adequate preparation of health professionals regarding child maltreatment	Yes	1,166	100
	No	0	0
Under-graduate training on concepts related to child maltreatment during undergraduate studies	Yes	1,034	88.68
	No	132	11.32
Lockdown and social distancing have had, or will have in the future, consequences on children's mental health	Strongly disagree	0	0
	Disagree	19	1.63
	Neutral	268	22.98
	Agree	388	33.28
	Strongly agree	491	42.11
**Focus on management of pediatric patients during COVID-19 pandemic**			
Knowledge of the concept of defensive medicine	Yes	860	73.76
	No	306	26.24
Pediatricians are more prone to defensive medicine	Strongly disagree	5	0.58
	Disagree	66	7.67
	Neutral	120	13.95
	Agree	466	54.19
	Strongly agree	203	23.60
Increased use of defensive medicine due to the pandemic	Yes	699	81.28
	No	161	18.72
Knowledge of the government's measures to protect vaccine doctors	Yes	509	43.65
	No	657	56.35
Implementation of protections for frontline and vaccination doctors	Yes	938	80.45
	No	228	19.55
Increase in medical-legal litigation	Yes	924	79.25
	No	242	20.75
Extension of vaccination to the pediatric population	Strongly disagree	0	0
	Disagree	23	1.97
	Neutral	295	25.32
	Agree	386	33.05
	Strongly agree	462	39.66
The government provided adequate information to parents about COVID-19 vaccination	Strongly disagree	48	4.12
	Disagree	239	20.52
	Neutral	405	34.76
	Agree	311	26.61
	Strongly agree	163	13.99
Telemedicine should be used in the management of all non-serious patients, including pediatric patients	Strongly disagree	48	4.12
	Disagree	216	18.52
	Neutral	391	33.53
	Agree	344	29.50
	Strongly agree	167	14.32
Management of non-serious patients using telemedicine has had or will have negative effects on their health	Strongly disagree	135	11.58
	Disagree	306	26.24
	Neutral	312	26.76
	Agree	326	27.96
	Strongly agree	87	7.46
Hospital policies have been adapted to the management of pediatric patients	Strongly disagree	0	0
	Disagree	42	3.61
	Neutral	357	30.56
	Agree	393	33.73
	Strongly agree	374	32.10
Intra-hospital isolation measures taken during the pandemic have had negative effects on children's mental health, or will have in the future	Strongly disagree	1	0.09
	Disagree	25	2.14
	Neutral	280	24.01
	Agree	420	36.02
	Strongly agree	440	37.74

### Focus on child maltreatment

The majority of students (52.06%) were familiar with child maltreatment situations; in addition, there was a strong concern among them that both the number of occurrences of child abuse has grown since the pandemic and that it may continue to rise (94.94 and 89.97%, respectively).

Contrarily, few students were aware of the government's initiatives to reduce this phenomenon (11.66%).

All students agreed on the fact that health professionals must be adequately prepared to recognize child maltreatment cases and, the majority of them believe it is advisable to further their training on this subject during their studies (100 and 88.68%, respectively).

Finally, the majority of them concurred that social distancing and lockdown have had harmful effects on children's mental health or would in the future (agree 33.28%; strongly agree 42.11%).

### Management of pediatric patients during COVID-19 pandemic

The majority of students were familiar with the concept of defensive medicine (73.76%); of the latter, approximately 78% agreed on the fact that pediatricians tend to be more prone to defensive medicine (agree: 54.19%; strongly agree: 23.60%) and that pediatric patients expose pediatricians to increased use of defensive medicine (81.28%).

Only 43.65% were aware of government measures to protect vaccine doctors, despite the fact that many believed measures should be implemented, and the majority of them are concerned because they believe there will be an increase in medical-legal litigation cases as a result of the pandemic (80.45 and 79.25%, respectively).

With regard to vaccination, many agreed that it should also be extended to pediatric patients (agree: 33.05%; strongly agree: 39.66%), though a minority agreed that parents should be adequately informed on the benefits, risks, and side effects of vaccines (agree: 26.61%; strongly agree: 13.99%).

Moreover, there was no agreement neither on the use of telemedicine for non-serious pediatric patients nor on the consequences it might have on their health.

Finally, although many of them agreed on the hospital management of pediatric patients during the pandemic (agree: 33.73%; strongly agree: 32.10%) many of them thought that such measures have had negative effects on children's mental health or will have in the future (agree: 36.02%; strongly agree: 37.74%).

###  Multivariable analysis

Table 3 summarizes the results of the multivariable analysis.

**Table 3:** Knowledge and attitudes on child maltreatment and management of pediatric patients during COVID-19 pandemic (adjusted *OR*, Odds Ratio; *CI*, 95% confidence interval).

		**Child maltreatment**		**OR (IC)**	**OR adjusted (IC)**
		**No (%)**	**Yes (%)**		
Gender	Male	257 (46.6)	294 (53.3)	–	
	Female	334 (54.3)	281 (45.7)	0.73 (0.58–0.92)	
Age		23 (22–24)	24 (22–25)	1.25 (1.17–1.33)	
Year of course	1–4	420 (61.8)	260 (38.2)	–	
	5–6	171 (35.2)	315 (64.8)	2.97 (2.33–3.79)	2.86 (1.98–4.13)
Area of residence	Center-North	238 (54.2)	201 (45.8)	–	
	South and Island	353 (48.6)	374 (51.4)	1.25 (0.98–1.59)	
		**Management of pediatric** **patients during** **COVID-19 pandemic**		**OR (IC)**	**OR adjusted (IC)**
		**No (%)**	**Yes (%)**		
Gender	Male	252 (45.7)	299 (54.3)	–	
	Female	304 (49.4)	311 (50.6)	0.86 (0.68–1.08)	
Age		22 (21–24)	24 (22–25)	1.47 (1.37–1.58)	1.33 (1.20–1.47)
Year of course	1–4	408 (60)	272 (40)	–	
	5–6	148 (30.4)	338 (69.6)	3.42 (2.67–4.38)	1.54 (1.07–2.22)
Area of residence	Center-North	213 (48.5)	226 (51.5)	–	
	South and Island	343 (47.2)	384 (52.8)	1.05 (0.83–1.33)	
Knowledge on child maltreatment	No	316 (53.5)	275 (46.5)	–	
	Yes	240 (41.7)	335 (58.3)	1.60 (1.27–2.02)	

A high level on knowledge on child maltreatment was significantly associated with the age of respondents (*p* < 0.0001), female gender (*p* = 0.00), clinical years of course (*p* < 0.001) and Southern regions of origin (*p* = 0.061). Testing the significant covariates in the regression, only the covariate on year of course remained statistically significant (*p* < 0.0001, adjusted OR 2.86 CI 95% [1.98–4.13]).

A high level of knowledge on the management of COVID-19 pandemic was significantly associated with the age (*p* < 0.001), clinical years of course (*p* < 0.0001) and knowledge on child maltreatment (*p* < 0.001). Testing the significant covariates altogether, age and clinical years of course remained significant (*p* < 0.001 adjusted OR 1.33 CI 95% [1.20–1.47] and *p* < 0.02 adjusted OR 1.54 CI 95% [1.07–2.22] respectively).

## Discussion

According to our findings, the majority of students are familiar with child maltreatment situations; additionally, furthermore, there is a strong concern among them both that cases have increased during the pandemic, and that they may increase in the future.

These results are consistent with earlier studies that showed how the COVID-19 pandemic has amplified dangerous conditions for children, families, and communities worldwide, despite the fact that no studies have ever been conducted to assess the perception of child maltreatment among undergraduate medical students (10–14).

However, it is important to emphasize that, in contrast to research done in the pre-pandemic period, medical students are more familiar with this topic, and that this knowledge is mostly tied to the year of the course to which they belong (15–17).

Indeed, after evaluating the significant variables in the regression, only the covariate on course year remained statistically significant on awareness of child maltreatment. These findings might be the fact that in the fifth and sixth years of the program, both the forensic medicine and pediatrics courses examine the risk factors of child abuse. Finally, students agree that these topics should both be known by all healthcare professionals and explored in depth during undergraduate studies.

For these reasons, we are convinced that additional seminars analyzing these topics from both a clinical and a medico-legal point of view during the degree course could be beneficial (18).

Regarding management of pediatric patients during the COVID-19 pandemic, most students that were familiar with the concept of defensive medicine agreed that pediatric patients expose pediatricians to an increased use of defensive medicine. In this regard, several studies have shown an increased risk of using defensive medicine to avoid the risk of medico-legal litigation among physicians; parental worry and anxiety, even for conditions that could be managed at home, may be the major contributing factors when dealing with pediatric patients. In such cases, unnecessary tests are also carried out with the aim of preventing the risk of complaints. It is important to acknowledge that even students, due to a lack of faith in insurance coverage, believe that defensive medicine could be a solution when dealing with pediatric patients; indeed, the lack of confidence in insurance coverage was identified as a key predictor of defensive medicine practice itself (19, 20).

On the other hand, some authors have demonstrated a greater rate of appropriateness of imaging studies during the pandemic, via a decrease in imaging investigations without evidence of bone fractures, thus implying a reduction in the use of defensive medicine (21).

In agreement with earlier research, many physicians, including vaccination doctors, are concerned about the rise in medical-legal lawsuits brought on by the epidemic (6).

This may also help explain why many physicians believe that the government's regulations for vaccination physicians should be enacted even if only a small percentage of people are aware of them (22).

A critical aspect also emerged from this survey: the lack of awareness of the legislative protections around the use of telemedicine for pediatric patients. In fact, there was no consensus about either the use for pediatric patients who weren't in a life-threatening situation or the potential effects on their health. This lack of agreement could be due to several factors, including the lack of understanding of the concept of telemedicine and the possibility that those from lower socioeconomic groups won't have access to treatment, leading to an increase in disparities (23).

In addition to this is the fact that legal issues may arise with informed consent, authorization, and accreditation profiles to the protection of patients' personal data; thus, as suggested by some authors, there is a need for governments to further develop legislations to ensure the safety of patients managed through the use of telemedicine (24, 25). Finally, a strong concern about the present and future impact of the lockdown and intra-hospital isolation measures on children's mental health emerged from the study. This strong concern is supported by evidence from the worldwide literature in addition to being perhaps related to how the lockdown affected students' mental health.

In fact, on the one hand, Nearchou et al. demonstrated how COVID-19 has had a negative impact on young people's mental health, which is primarily characterized by depression and anxiety, on the other hand, Villani et al., in a study aimed at assessing the psychological impact of the pandemic on undergraduate students, showed that 35.33% of University students had symptoms of anxiety and 72.93% of depression, although with mild symptoms (26–28).

For these reasons, it could be beneficial to establish as many mental health support services oriented on offering measures for supporting healthy coping mechanisms throughout the current crisis as possible. The strengths and weaknesses of this study must be taken into account. Because enrollment is restricted to a single University, it is possible that it may not be accurately representative of the medical and surgical student population in Italy. This study, however, is the first to examine the perceptions, understanding of, and attitudes of medicine and surgery students toward themes of medico-legal importance in the context of the COVID-19 epidemic, with an emphasis on child safety and child maltreatment. It could offer a practical instrument to approach these subjects in training sessions and educational initiatives.

## Conclusions

Our study showed that medicine and surgery students are familiar with the concepts of child maltreatment, child safety, and medical liability regarding the management of pediatric patients during COVID-19 pandemic.

This knowledge is mainly implemented by deepening these concepts during the undergraduate studies since a high level of knowledge on child maltreatment and on the management of COVID-19 pandemic was significantly associated with clinical years of course.

In addition, low level of knowledge of telemedicine, legislative protections concerning minors, and those concerning vaccination doctors emerged.

For these reasons, we believe that specific seminars analyzing these concepts should be included in the study plan, as well as multidisciplinary seminars held by forensic scientists, pediatricians, and social workers in order to raise awareness on the phenomenon of child maltreatment also among “future doctors”.

## Data availability statement

The raw data supporting the conclusions of this article will be made available by the authors, without undue reservation.

## Ethics statement

The studies involving human participants were reviewed and approved by the Ethics Committee of the Policlinico Universitario A. Gemelli IRCCS. The patients/participants provided their written informed consent to participate in this study.

## Author contributions

GA and AO designed, conducted, and wrote the paper. FB conducted part of the statistical part of the article. All authors reviewed and approved the draft of the manuscript and ensured the accuracy and integrity of the work before submission. All authors contributed to the article and approved the submitted version.

## Funding

Funds were from Linea D1, Università Cattolica del Sacro Cuore (recipient: AO).

## Conflict of interest

The authors declare that the research was conducted in the absence of any commercial or financial relationships that could be construed as a potential conflict of interest.

## Publisher's note

All claims expressed in this article are solely those of the authors and do not necessarily represent those of their affiliated organizations, or those of the publisher, the editors and the reviewers. Any product that may be evaluated in this article, or claim that may be made by its manufacturer, is not guaranteed or endorsed by the publisher.
