# Supplementary material for: Child maltreatment and management of pediatric patients during COVID-19 pandemic: Knowledge, awareness, and attitudes among students of medicine and surgery. A survey-based analysis
Source: Front Public Health. 2022 Sep 20;10:968286. doi: 10.3389/fpubh.2022.968286 (PMC9531710; doi:10.3389/fpubh.2022.968286)
Supplement: Supplementary file 1 [file Table_1.DOCX]

**Supplementary file 1**

1. Gender: Male/Female

2. Year of birth

3. Year of course: 1/2/3/4/5/6

4. Region of origin: Abruzzo/Basilicata/Calabria/Campania/Emilia-Romagna/Friuli Venezia Giulia/Latium/Liguria/Lombardy/Marches/Molise/Piedmont/Apulia/Sardinia/Sicily/Tuscany/Trentino Alto-Adige/Umbria/Aosta Valley /Veneto

5. Are you aware of the situations that constitute child maltreatment? Yes/No

6. Do you think there was an increase in child maltreatment cases during the pandemic? Yes/No

7. Do you think the pandemic may lead in the future to an increase in cases of child maltreatment

Children? Yes/No

8. Do you know of measures to prevent the increase in cases of maltreatment children instituted by the government? Yes/No

9. Do you think that health professionals who are in the care of the child should be adequately trained to recognize cases of child maltreatment? Yes/No

10. Do you think that during your undergraduate studies, the topic of child maltreatment

should be covered in depth and in different integrated courses? Yes/No

11. Lockdown and social distancing have had, or will have in the future, consequences

on the mental health of children. Strongly disagree/Disagree/Neutral/Agree/Strongly agree

12. Do you know the concept of defensive medicine? Yes/No

13. If yes, you agree with the statement Pediatricians are more prone to defensive medicine. Strongly disagree/Disagree/Neutral/Agree/Strongly agree

14. Do you think the pandemic has increased the use of defensive medicine? Yes/No

15. Do you know government’s measures to protect vaccine doctors? Yes/No

16. Do you think the state should implement protections against physicians who have dealt with vaccination? Yes/No

17. Do you think the pandemic has increased the risk of medico-legal litigation or may

do so in the future? Yes/No

18. Do you agree with the government's choice regarding the expansion of anti

covid-19 to the pediatric population as well? Strongly disagree/Disagree/Neutral/Agree/Strongly agree

19. The government provided adequate information to parents about covid-19 vaccination Strongly disagree/Disagree/Neutral/Agree/Strongly agree

20. Telemedicine should be used in the management of all non-serious patients, including

pediatric ones Strongly disagree/Disagree/Neutral/Agree/Strongly agree

21. Management of non-serious patients using telemedicine has had or will have negative effects on their health. Strongly disagree/Disagree/Neutral/Agree/Strongly agree

22. Hospital policies have been adapted to the management of pediatric patients. Strongly disagree/Disagree/Neutral/Agree/Strongly agree

23. Intra-hospital isolation measures taken during the pandemic have had

negative effects on children's mental health, or will in the future Strongly disagree/Disagree/Neutral/Agree/Strongly agree
